# Sildenafil reduced neuroinflammation and improved white matter injury in a rat model of term neonatal hypoxic-ischemic encephalopathy

**DOI:** 10.1038/s41598-025-34307-6

**Published:** 2025-12-31

**Authors:** Armin Yazdani, Virginie Bleau, Ruofan Song, Yandi Zheng, Palig Balian, Zehra Khoja, Mathilde Chevin, Pia Wintermark

**Affiliations:** 1 Research Institute of the McGill University Health Centre, 1001 boul. Décarie, Site Glen Block E, EM0.3244, Montreal, QC H4A 3J1 Canada; 2 Division of Newborn Medicine, Department of Pediatrics, Montreal Children’s Hospital, Montreal, Canada

**Keywords:** Brain, Hypoxia–ischemia, mTOR pathway, Neonatal encephalopathy, Neuroinflammation, Neurorestoration, Newborn, Oligodendrogenesis, White matter injury, Diseases, Neurology, Neuroscience

## Abstract

Neonatal hypoxic-ischemic encephalopathy (HIE) can cause lifelong neurological impairments. In its tertiary phase, ongoing neuroinflammation creates a toxic environment that promotes neuronal and oligodendrocyte loss. Sildenafil has shown neuroprotective effects in adult models by reducing inflammation and supporting oligodendrocyte survival, but its role in HIE remains unexplored. This study investigated the effects of sildenafil on neuroinflammation and white matter injury in a rat model of term neonatal HIE. Hypoxia–ischemia (HI) was induced in postnatal day 10 (P10) male Long-Evans rats via a left carotid ligation followed by 2 h of hypoxia (8% oxygen). Pups were randomized to receive oral sildenafil or vehicle starting 12 h post-HI, twice daily for 7 days. White matter integrity (corpus callosum and external capsule), oligodendrocyte presence, and glial activation were assessed by histology and immunohistochemistry. Inflammatory markers were measured by enzyme-linked immunosorbent assay (ELISA), and signaling pathways were examined by Western blot. Outcomes were compared to sham and untreated HI controls. HI significantly increased number of GFAP + reactive astrocytes and Iba1 + microglia, alongside elevated TNFα and IL-1β levels. Thickness of the corpus callosum and left external capsule was reduced. Sildenafil treatment — particularly at medium and high doses — attenuated astrocytes and microglia activation, restored microglial morphology, and normalized cytokine expression. White matter thickness was significantly improved, with increased numbers of total Olig2 + and mature CC1 + oligodendrocytes. Mechanistically, sildenafil restored p-AKT levels, which suggests involvement of the PI3K/AKT/mTOR pathway. Sildenafil significantly reduced neuroinflammation, improved white matter integrity, and supported oligodendrocyte recovery after neonatal HI. These findings highlight the potential of sildenafil as a neurorestorative therapy during the tertiary phase of injury in neonatal HIE.

## Introduction

Neonatal hypoxic-ischemic encephalopathy (HIE) due to birth asphyxia remains a leading cause of neonatal death and long-term neurological disability affecting approximately 3 per 1000 live births worldwide^1^. Therapeutic hypothermia (TH), initiated within 6 h of life and for 72 h, is the only proven treatment for HIE and reduces mortality and neurodevelopmental impairments^2–4^. However, TH is not effective for all affected neonates and offers no neurorestorative properties^3^. A critical need exists for adjunct therapies that promote brain *repair* (“neurorestorative” therapies) after an initial injury.

HIE evolves in three phases. The initial phase (0–6 h) involves energy failure and necrosis^5^. The secondary phase (6–48 h) triggers apoptosis through excitotoxicity, oxidative stress, and mitochondrial damages^6,7^. The tertiary phase (beyond 48 h) is marked by chronic neuroinflammation and epigenetic changes that impair recovery and lead to further neuronal and oligodendrocyte loss through apoptosis and autophagy^8^. Persistent glial activation and inflammatory cytokine release disrupt essential neurodevelopmental processes such as neurogenesis, oligodendrogenesis, and synaptogenesis^9–14^. While TH targets the early phases of injury, therapies that address the tertiary phase may enhance long-term recovery.

Sildenafil (Viagra®), a phosphodiesterase type 5 (PDE5) inhibitor that increases intracellular cGMP, shows promise as a neurorestorative therapy. It has been found to enhance both functional and structural recovery in adult stroke models^15,16^ and is already used safely in neonates with persistent pulmonary hypertension^17,18^. In a rat model of term neonatal HIE, sildenafil enhanced brain and retinal function and structure after hypoxia–ischemia^19–21^ and increased neuronal density near injury sites^19^. However, its effects on neuroinflammation and white matter injury in this model remain unclear. In adult models of neurodegenerative disease, sildenafil has been shown to reduce neuroinflammation^22–27^ and promote white matter repair by supporting oligodendrocyte survival^23,28,29^. The present study aimed to evaluate the effects of sildenafil on neuroinflammation and white matter injury in a rat model of term HIE.

## Material and methods

### Animals

All procedures followed institutional standard operating procedures (SOPs) and the Canadian Council on Animal Care’s (CCAC) guidelines in accordance with the Animals for Research Act, were approved by the local Animal Care Committee, and were reported in accordance with ARRIVE guidelines^30^. Adult female Long-Evans rats male-only litters (Harlan Laboratories) were housed under standard conditions with food and water ad libitum. Pups remained with their mother until weaning at postnatal day 21 (P21). Daily monitoring was performed by facility technicians and researchers.

### Induction of term neonatal HIE

To model brain injury in term human neonates with HIE^31–36^, we used the well-established Vannucci model^31–34,37,38^. On P10, rat pups underwent left carotid artery ligation (ischemia) followed by 2 h of hypoxia (8% oxygen)^19^. Sham-operated controls underwent identical procedures without hypoxia–ischemia (HI), and sham pups were kept normothermic using warming blankets (Cincinnati Sub-Zero, Cincinnati, USA) during separation from dams. Sample size (n = 6–12 per group) was based on feasibility, ethical constraints, and the 3Rs principle (replacement, reduction, refinement).

### Sildenafil preparation and administration

Sildenafil (Viagra®, Pfizer; 100-mg tablets) was prepared as previously described^19,20^. From 12 h post-HI, we randomized pups to receive sildenafil or vehicle by oral gavage twice daily for 7 days (P10-17). Doses in HI pups included 2, 10, and 50 mg/kg for histology/immunohistochemistry, and 50 mg/kg for Western blot/ELISA, based on human-equivalent dosing^39^ and previous efficacy studies in cortex/retina^19,20^.

### Histology

We collected brains at P30^19,20^ and sectioned them at –2.16 mm from Bregma. We imaged hematoxylin and eosin-stained coronal sections using a Leica DM4000B microscope and a Leica DFC450C digital camera (Leica DFC450C, Leica Microsystems, Wetzlar, Hessen, Germany). We stitched overlapping 5 × images using a Microsoft Image Composite Editor. Using ImageJ (Image Processing and Analysis in Java)^40^, we blindly measured the thicknesses of the corpus callosum and ipsilateral external capsule on two sections per animal and averaged them.

### Immunohistochemistry

**Fig. 1:**
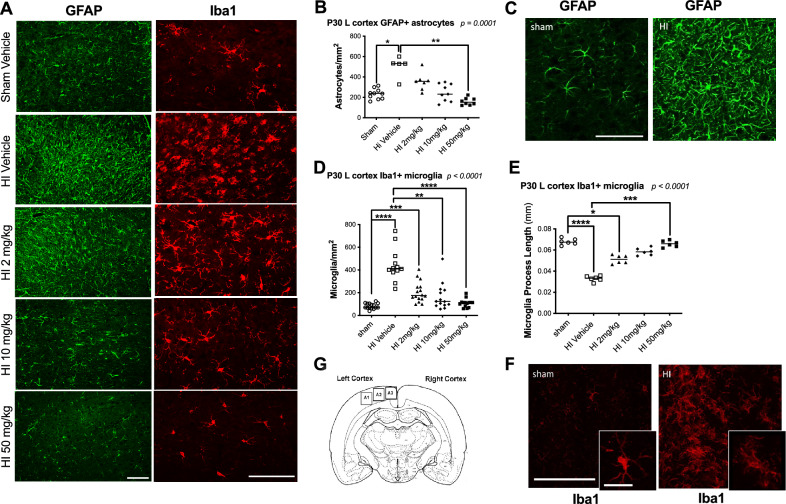
Immunostaining of inflammatory cells in the ipsilateral (left) cortex of sham and HI-exposed rat pups treated with sildenafil or vehicle. (**A**) Representative immunofluorescent micrographs showing GFAP + reactive astrocytes (green) and Iba1 + activated microglia (red) in the ipsilateral cortex near the infarct boundary zone. Scale bar = 100 µm. (**B**) Quantification of GFAP + reactive astrocytes in three fields of view in the ipsilateral cortex near the infarct boundary zone. Scale bar = 100 µm. Data are presented as median with individual data points representation. (**C**) Representative magnified immunofluorescent micrographs of GFAP + astrocytes demonstrating a reactive morphology. Scale bar = 100 µm. (**D**) Quantification of Iba1 + microglia in three fields of view in the ipsilateral cortex near the infarct boundary zone (median with individual data points representation). (**E**) Quantification of microglial process length in three fields of view in the ipsilateral cortex near the infarct boundary zone (median with individual data points representation). (**F**) Representative magnified immunofluorescent micrographs of Iba1 + activated microglia demonstrating morphology. Scale bar = 100 µm; inset (zoom) scale bar = 10 µm. (**G**) Schematic of ipsilateral cortical zone near infarct boundary (A1–A3) where astrocytes and microglia were counted. Statistical significance was determined using Kruskal–Wallis tests with Dunn’s post hoc comparisons: *p < *0.05*, **p < *0.01*, ***p < *0.001,* ***p < *0.0001*. Number of animals: 10–15 sham, 5–12 HI vehicle, 7–16 HI 2 mg/kg, 9–14 HI 10 mg/kg, and 8–14 HI 50 mg/kg.

**Fig. 2:**
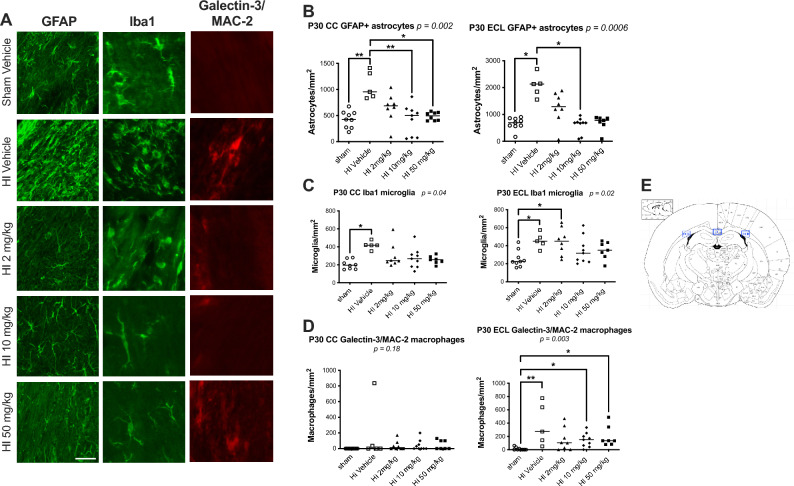
Immunostaining of inflammatory cells in the white matter of sham and HI-exposed rat pups treated with sildenafil or vehicle. (**A**) Representative immunofluorescent micrographs of the GFAP + reactive astrocytes (green), Iba1 + activated microglia (green), and Galectin-3/Mac2 + phagocytic macrophages (red) in the ipsilateral left external capsule (ECL). Scale bar = 30 µm. (**B**–**D**) Quantification of (**B**) GFAP + astrocytes, (**C**) Iba1 + microglia, and (**D**) Galectin-3/Mac2 + macrophages in a single field of view within the corpus callosum (CC) and in the ipsilateral ECL. Data are presented as median with individual data points representation. (**E**) Schematic of CC and ECL zones where astrocytes, microglia and macrophages were counted. Statistical significance was assessed using the Kruskal–Wallis test with Dunn’s posthoc comparison tests: *p < *0.05*, **p < *0.01*. Number of animals: 8–9 sham, 5 HI vehicle, 8 HI 2 mg/kg, 9 HI 10 mg/kg, and 7–8 HI 50 mg/kg.

At P30, we initially incubated the sections in a universal antigen retrieval reagent at 95 °C for 20 min, and then blocked them in phosphate-buffered saline containing tween20 and gelatin (PBS-GT). We incubated the primary antibodies overnight at 4 °C in PBS-GT. We used the markers glial fibrillary acidic protein (GFAP) (mouse anti-GFAP, SMI-22R; Covance, Princeton, New Jersey, USA; dilution 1:1000, incubation overnight at 4 °C) for astrocytes, ionized calcium binding adaptor molecule 1 (Iba1) (rabbit anti-Iba1, 019–19,741; Wako Pure Chemical Industries, Osaka, Japan; dilution 1:600, incubation overnight at 4 °C) for microglia, Galectin-3/MAC-2 (mouse anti-Galectin-3/MAC-2, ab2785; Abcam, Cambridge, UK; dilution 1:1000, incubation overnight at 4 °C) for activated macrophages, anti-oligodendrocyte transcription factor (Olig2) (rabbit anti-Olig2, ab109186; Abcam, Cambridge, UK; dilution 1:500, incubation overnight at 4 °C) for total oligodendrocytes, and adenomatous polyposis coli (CC1) (mouse anti-CC1, ab16794; Abcam, Cambridge, UK; dilution 1:1000, incubation overnight at 4 °C) for mature oligodendrocytes. We also used the appropriate AlexaFluor® or Cy™3 secondary antibodies. We counterstained the sections with DAPI and mounted them with Vectashield (H1200; Vector Laboratories, Burlingame, CA, USA). We performed the imaging using a fluorescent microscope (20x). For quantitative measurements, coronal brain sections were selected at a consistent anatomical level characterized by visible hippocampi, reduced lateral ventricles, a visible third ventricle, and clear delineation of the thalamus (Figs. 1G and 2E). Stained brain sections were imaged using a fluorescent microscope (Leica DM4000B LED, Leica Microsystems, Wetzlar, Germany) with a 20 × objective. For each animal, 2–3 fields were captured in the left (ipsilateral) cortex of HI animals near the infarct boundary zone and extending toward the midline (Fig. 1G); corresponding cortical fields were captured in sham-vehicle animals at matched anatomical landmarks to ensure valid comparisons across groups. Additional 20 × micrographs were also obtained from the corpus callosum (CC) and the left (ipsilateral) external capsule (ECL) to evaluate white matter, using identical anatomical reference points across animals; the CC was measured at the midline between hemispheres, and the ECL on the left side at the same coronal level (Fig. 2E). Image analysis was performed using an image analysis software (ImageJ) (Image Processing and Analysis in Java)^40^; after converting the scale of the original pictures in mm^2^, we quantified the number of astrocytes (GFAP), microglia (Iba1), macrophages (galectin-3/Mac-2), and oligodendrocytes (Olig2, CC1, Olig2 + /CC1-). We also assessed the microglial activation by measuring the maximal process length from five randomly selected microglia cells per animal. One investigator took pictures of the respective fields of view and another blinded investigator, performed the cell counts.

### Western blotting

For western blotting, we euthanized the rats at P12, P17, and P30. The middle third of the ipsilateral cortex and white matter (excluding basal ganglia, hippocampus, and cerebellum) was flash frozen. We lysed the tissues by sonication in an ice-cold RIPA buffer (89,901, ThermoFisher Scientific, Waltham, Massachusetts, USA) with protease inhibitors (S8820, Sigma-Aldrich, St. Louis, Missouri, USA), centrifuged (12,000 RCF, 10 min, 4 °C), and measured the protein concentration (BCA assay) (23,225; Thermo Fisher Scientific, PierceTM, Waltham, Massachusetts, USA). We loaded equal protein amounts on SDS–polyacrylamide CriterionTM TGXTM precast gels (567–1085; Bio-Rad®, Hercules, California, USA) and then transferred them to a Polyvinylidene difluoride (PVDF) membrane (10,600,023; AmershamTM HybondTM, Boston, Massachusetts, USA). We blocked the membranes with 5% dried non-fat milk in Tris-buffered saline containing 0.1% Tween-20 (9005-64-5; Fisher BioReagentsTM, Hampton, New Hampshire, USA), and then incubated them overnight at 4 °C with the following primary antibodies**:** cleaved poly-ADP-ribose polymerase (PARP) (rabbit anti-PARP, 9542; Cell Signaling Technology, Danvers, Massachusetts, USA; dilution 1:1000), a marker of apoptosis; superoxide dismutase 1 (SOD1) (rabbit anti-SOD1, ab13498; Abcam, Cambridge, UK; dilution 1:1000), a marker of oxidative stress; synaptophysin (rabbit anti-synaptophysin, 5461; Cell Signaling Technology, Danvers, Massachusetts, USA; dilution 1:1000), a marker of synaptic density; oligodendrocyte lineage transcription factor 2 (Olig2) (mouse anti-Olig2, MABN50; Sigma-Aldrich, St. Louis, Missouri, USA; dilution 1:2000), a marker of total oligodendrocytes; neuronal nuclei antibody (NeuN) (mouse anti-NeuN, MAB377; Millipore, Burlington, Massachusetts, USA; dilution 1:500), a marker of mature neurons; and phosphorylated protein kinase B (pAKT) (rabbit anti-phospho-Akt Ser473 D9E, 4060; Cell Signaling Technology, Danvers, Massachusetts, USA; dilution 1:1000), an upstream regulator of the mechanistic target of rapamycin (mTOR) pathway. We used chemiluminescence detection and an imaging system (Amersham Imager 600, General Electric, Boston, Massachusetts, USA), and we quantified band intensities using Image Lab® software (Bio-Rad®, Hercules, California, USA), normalized to ß-actin (mouse anti-ß-actin; Millipore Sigma, Oakville, Ontario, Canada; dilution: 1:5000).

### Enzyme-linked immunosorbent assay (ELISA)

We measured pro-inflammatory cytokines Interleukin 1 beta (IL-1β) and tumor necrosis factor alpha (TNFα) and anti-inflammatory cytokine interleukin-1 receptor antagonist (IL-1RA) in the ipsilateral cortex at P12, P17, and P30 using ELISA kits (respectively, RTA00 and RLB00; R&D Systems, Minneapolis, Minnesota, USA; P25086; RayBiotech, Peachtree Corners, Georgia, USA) per manufacturer instructions.

### Data analysis

We randomly assigned pups to treatment groups. We made group comparisons using Kruskal–Wallis non-parametric tests, followed by Dunn’s post hoc tests to adjust the α–level as necessary. We considered a p-value < *0.05* to be statistically significant. We conducted analyses using GraphPad Prism® (GraphPad Software Inc., San Diego, CA, USA).

## Results

### Sildenafil treatment following HI reduced activation of inflammatory cells in both the cortex (near the infarct boundary zone) and the white matter, along with decreased levels of pro-inflammatory cytokines

**Fig. 3:**
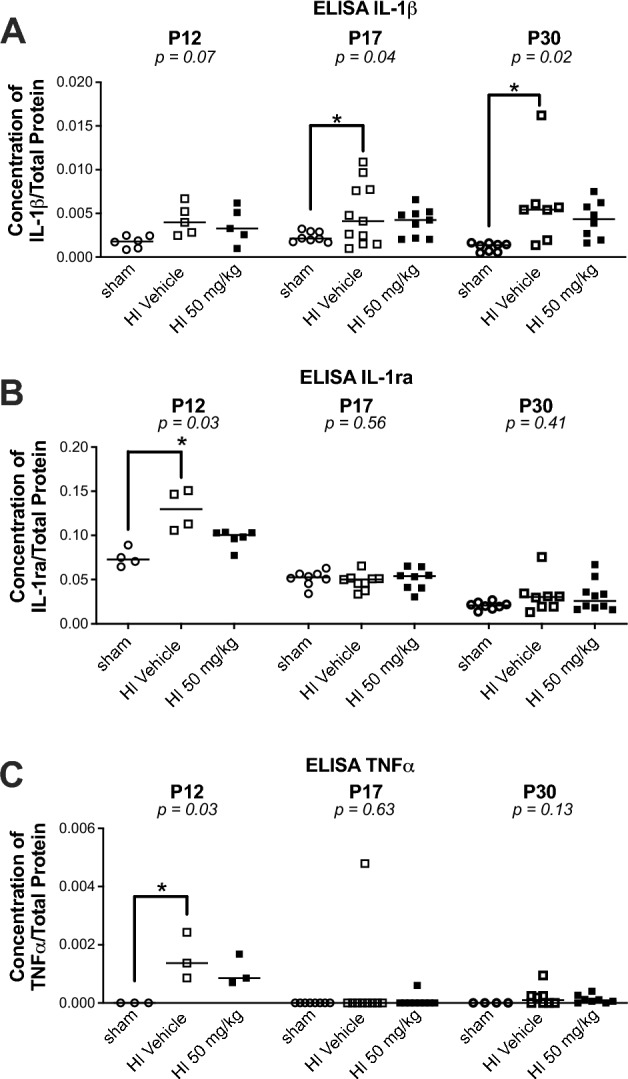
ELISA analysis of cytokine levels in the ipsilateral (left) cortex and white matter of sham and HI-exposed rat pups treated with sildenafil or vehicle. (**A**–**C**) Cytokines concentrations normalized to total protein content at P12, P17 and P30: (**A**) IL-1β; (**B**) IL-1ra; (**C**) TNFα. Data are presented as median with individual data points representation. Statistical significance was determined using the Kruskal–Wallis test with Dunn’s posthoc comparison tests: *p < *0.05*. Number of animals: P12: 3–6 sham, 3–5 HI vehicle, 3–6 HI 50 mg/kg; P17: 8 sham, 9–11 HI vehicle, 8–9 HI 50 mg/kg; P30: 4–8 sham, 7–8 HI vehicle, 8–10 HI 50 mg/kg.

At P30, HI significantly increased the number of GFAP + reactive astrocytes in the ipsilateral cortex (median [interquartile ranges]: 533 cells/mm^2^ [529–601], *p* < *0.05*) (Fig. 1), corpus callosum (954 [852–1362], *p* < *0.01*) (Fig. 2), and external capsule (2133 [1850–2691], *p* < *0.05*) (Fig. 2), compared to sham (cortex: 239 [187–275]; CC: 425 [269–553]; ECL: 692 [576–850]). HI also elevated Iba1 + microglia in the cortex (413 [383–506], *p* < *0.0001*] (Fig. 1), CC (420 [352–482], *p* < *0.05*] (Fig. 2), and ECL (450 [384–533], *p* < *0.05*] (Fig. 2), versus sham (cortex: 70 [67–105]; CC: 195 [152–261], and ECL: 225 [188–318]) (Fig. 1). Microglial process length was reduced after HI (0.033 mm [0.031–0.035] vs sham: 0.068 mm [0.066–0.071], *p* < *0.0001*] (Fig. 1). Galectin-3/MAC-2 + macrophages were significantly increased in the ECL (273 [96–706] vs. 0 [0–27], *p* < *0.05*) (Fig. 2), but not in the CC (0 [0–435] vs. 0 [0–0]). Pro-inflammatory TNFα levels rose significantly at P12 (*p* < *0.05*), and IL-1β levels were elevated at P17 and P30 (*p* < *0.05*) (Fig. 3). IL-1ra was transiently increased at P12 (*p* < *0.05*), and then normalized (Fig. 3).

Sildenafil (all doses) reduced GFAP + astrocytes in all regions to levels no longer significantly different from sham (cortex: low-dose: 358 [281–375], medium-dose: 232 [164–337], and high-dose: 173 [140–197]; CC: low-dose: 687 [527–811], medium-dose: 500 [77–614], and high-dose: 495 [402–558]; ECL: low-dose: 1292 [973–1747], medium-dose: 689 [401–768], and high-dose: 781 [627–863]). Medium and high dose sildenafil had the strongest effect (cortex: high-dose: *p* < *0.01* vs. HI; CC: medium-dose: *p* < *0.01* vs. HI; high-dose: *p* < *0.05* vs. HI; ECL: medium-dose: *p* < *0.05* vs. HI). Similarly, medium and high doses significantly reduced Iba1 + microglia in all regions to sham levels (cortex: low-dose: 176 [147–252], *p* < *0.001* vs. sham; medium-dose: 123 [90–225], *p* < *0.01* vs. HI; and high-dose: 114 [79–116], *p* < *0.0001* vs. HI; CC: low-dose: 246 [223–369], medium-dose: 267 [178–327], and high-dose: 257 [227–290]; ECL: low-dose: 451 [303–590], *p* < *0.05* vs. sham, medium-dose: 318 [227–470], and high-dose: 350 [281–436]) and restored microglial process length in the cortex (low-dose: 0.051 mm [0.048–0.055], *p* < *0.05* vs. sham; medium-dose: 0.058 mm [0.055–0.062]; 0.066 mm [0.063–0.069], *p* < *0.001* vs. HI) (Figs. 1 and 2). Galectin-3 + macrophages in the ECL were reduced by all doses, although only the low-dose brought levels to those of sham (low-dose: 103 [7–314]; medium-dose: 150 [52–225], *p* < *0.05* vs. sham; and high-dose: 134 [81–378], *p* < *0.05* vs. sham) (Fig. 2). Sildenafil normalized TNFα and IL-1ra levels, respectively at P12, P17 and P30. IL-1β remained slightly elevated with sildenafil but without statistical significance.

### Sildenafil improved white matter thickness and promoted oligodendrogenesis

**Fig. 4:**
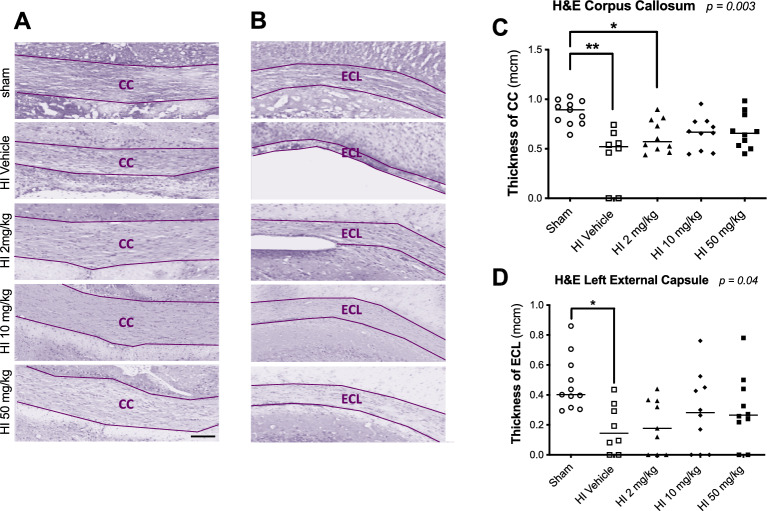
Hematoxylin and eosin (H&E) staining of coronal brain sections from sham and HI-exposed rat pups treated with sildenafil or vehicle. (**A**,**B**) Representative H&E-stained coronal sections at the level of the hippocampus showing (**A**) the corpus callosum (CC) and (**B**) the ipsilateral left external capsule (ECL). Scale bar = 100 µm. (**C**,**D**) Quantification of the thickness of (**C**) the CC and (**D**) the ECL. Data are presented as median with individual data points representation. Statistical significance was assessed using the Kruskal–Wallis test with Dunn’s posthoc comparison tests: *p < *0.05*, **p < *0.01*. Number of animals: 11 sham, 10 HI vehicle, 10 HI 2 mg/kg, 10 HI 10 mg/kg, and 8 HI 50 mg/kg.

**Fig. 5:**
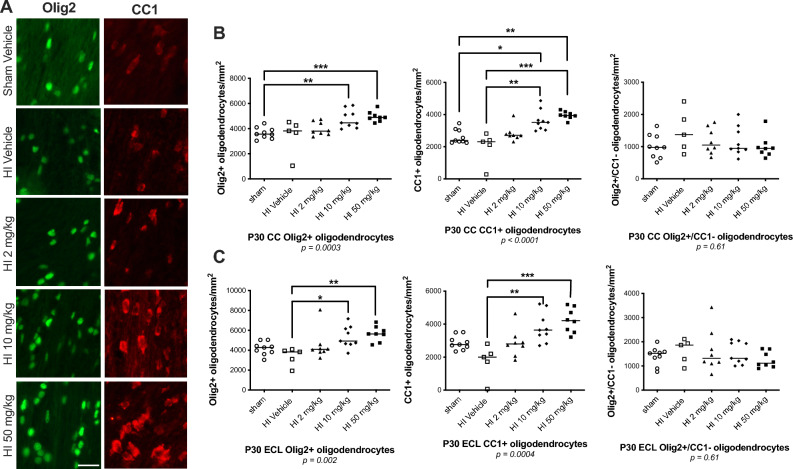
Immunostaining of oligodendrocytes in the white matter of sham and HI-exposed rat pups treated with sildenafil or vehicle. (**A**) Representative immunofluorescent micrographs of the total Olig2 + oligodendrocytes and mature CC1 + oligodendrocytes in the ipsilateral left external capsule (ECL). Scale bar = 30 µm. (**B**,**C**) Quantification of total Olig2 + oligodendrocytes, mature CC1 + oligodendrocytes and immature Olig2 + /CC1– oligodendrocytes were counted in one field of view in (**B**) the corpus callosum (CC) and (**C**) the ECL. Data are presented as median with individual data points representation. Statistical significance was assessed using the Kruskal–Wallis test with Dunn’s posthoc comparison tests: *p < *0.05*, **p < *0.01*, ***p < *0.001*. Number of animals: 9 sham, 5 HI vehicle, 8 HI 2 mg/kg, 9 HI 10 mg/kg, and 8 HI 50 mg/kg.

HI reduced thickness of the CC (median [interquartile ranges]: 0.52 µm [0.12–0.63], *p* < *0.01*) and ECL (0.14 [0.02–0.33], *p* < *0.05*) compared to sham (CC: 0.89 [0.75–0.98]; ECL: 0.40 [0.32–0.60]) (Fig. 4). No significant differences in Olig2 + or CC1 + oligodendrocytes were observed at baseline between HI (CC: Olig2 + : 3820 cells/mm^2^ [2367–4387]; CC1 + : 2314 [1202–2613]; ECL: Olig2 + : 3833 [2516–4013]; CC1 + : 2000 [896–2505]) and sham (CC: Olig2 + : 3561 [3283–3996]; CC1 + : 2391 [2288–3061]; ECL: Olig2 + : 4273 [3714–4734]; CC1 + : 2837 [2581–3504]) (Fig. 5).

Medium- and high-dose sildenafil significantly increased thickness of the CC (medium-dose: 0.68 [0.47–0.78]; high-dose: 0.64 [0.52–0.87]) and ECL (medium-dose: 0.28 [0.00–0.47]; high-dose: 0.27 [0.16–0.46]), restoring it to sham levels. Low dose also improved thickness of the ECL (0.18 [0.00–0.36]) but was less effective in the CC (0.57 [0.49–0.80], *p* < *0.05* vs. HI) (Fig. 4). In the CC, medium- and high-dose sildenafil increased total Olig2 + oligodendrocytes (low-dose: 3798 [3571–4601]; medium-dose: 4462 [4153–5463], *p* < *0.01* vs. sham; high-dose: 4889 [4655–5202], *p* < *0.001* vs. sham) and mature CC1 + oligodendrocytes (low-dose: 2710 [2608–2868]; medium-dose: 3513 [3094–4024], *p* < *0.05* vs. sham, *p* < *0.01* vs. HI; high-dose: 3945 [3851–4148], *p* < *0.001* vs. sham, *p* < *0.001* vs. HI). Similar effects were seen in the ECL (Olig2 + : low-dose: 4087 [3846–4484]; medium-dose: 4923 oligodendrocytes/mm^2^ [4630–6250], *p* < *0.05* vs. HI; high-dose: 5640 oligodendrocytes/mm^2^ [4739–6351], *p* < *0.01* vs. HI); CC1 + : mature oligodendrocytes (low-dose; 2798 [2218–3155]; medium-dose: 3641 oligodendrocytes/mm^2^ [3077–4688], *p* < *0.01* vs. HI; high-dose: 4210 oligodendrocytes/mm^2^ [3543–5006], *p* < *0.001* vs. HI) (Fig. 5). There was no difference between experimental groups in immature Olig2 + /CC1- oligodendrocytes.

### Sildenafil normalized apoptosis, antioxidant defense, and neuronal and synaptic markers altered by HI

**Fig. 6:**
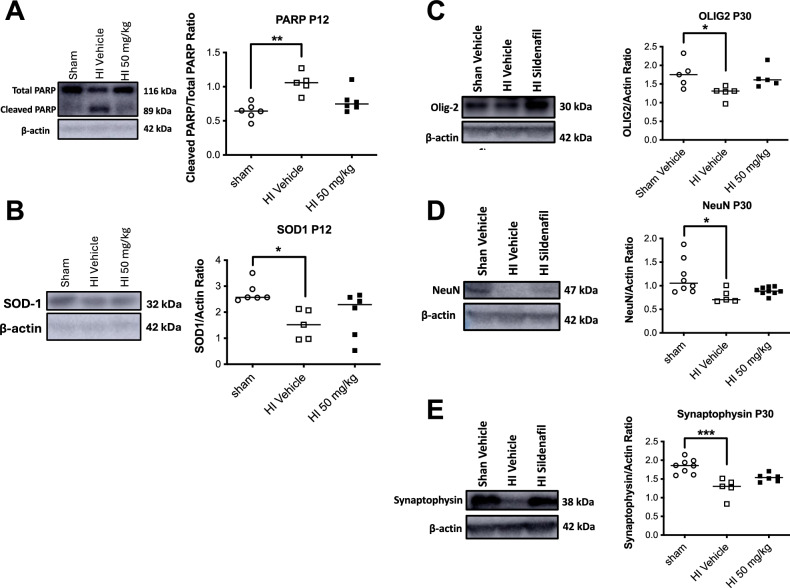
Immunoblots showing protein expression of markers for apoptosis, oxidative stress, oligodendrocytes, neurons and synapses markers in the cortex and white matter of HI and sham rat pups treated with sildenafil or vehicle. (**A**) Cleaved PARP, an apoptosis marker. (**B**) SOD1, a marker of oxidative stress. (**C**) Olig2, a marker of total oligodendrocyte lineage cells. (**D**) NeuN, a marker of mature neurons. (**E**) Synaptophysin, a synaptic density marker. Data are presented as median with individual data points representation. Statistical significance was assessed using the Kruskal–Wallis test with Dunn’s posthoc comparison tests: *p < *0.05*, **p < *0.01*, ***p < *0.001*. Number of animals: P12: 6 sham, 5 HI vehicle, 6 HI 50 mg/kg; P30: 5–8 sham, 5 HI vehicle, 5–9 HI 50 mg/kg.

HI caused a significant increase in cleaved PARP) levels at P12 in the ipsilateral cortex and white matter compared to sham rats (*p* < *0.01*) (Fig. 6). This increase was accompanied by a significant reduction in the antioxidant protein SOD1 (*p* < *0.05*) and a marked decrease in NeuN expression at P30 (*p* < *0.05*) (Fig. 6). Synaptophysin levels also were reduced significantly following HI (*p* < *0.001*) (Fig. 6).

Sildenafil treatment restored cleaved PARP levels to values no longer significantly different from sham at P12 (Fig. 6). SOD1 levels also returned to baseline with sildenafil treatment, indicating a reversal of HI-induced oxidative stress (Fig. 6). Similarly, NeuN and synaptophysin expression at P30 were normalized following sildenafil administration (Fig. 6).

### Sildenafil may reduce neuroinflammation through mTOR pathway activation

**Fig. 7:**
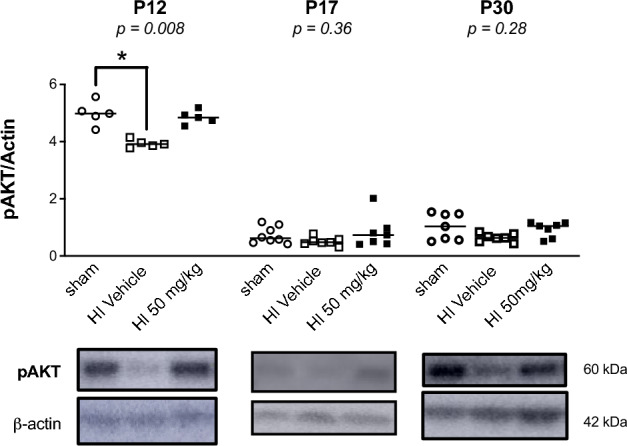
Immunoblot analysis of phosphorylated protein kinase B (pAKT), an upstream regulator of the mechanistic target of rapamycin (mTOR) pathway, in the cortex and white matter of HI and sham rat pups treated with sildenafil or vehicle. Data are presented as median with individual data points representation. Statistical significance was assessed using the Kruskal–Wallis test with Dunn’s posthoc comparison tests: *p < *0.05*. Number of animals: P12: 5 sham, 5 HI vehicle, 5 HI 50 mg/kg; P17: 8 sham, 7 HI vehicle, 7 HI 50 mg/kg; P30: 7 sham, 7 HI vehicle, 7 HI 50 mg/kg.

pAKT levels were decreased significantly at P12 following HI (*p* < *0.05*) compared to sham animals but returned to baseline levels after sildenafil treatment (Fig. 7).

## Discussion

In our rat model of term neonatal HIE, HI induced an increase in reactive astrocytes and microglia in the cortex adjacent to the infarct boundary, as well as in the white matter, which remained evident 20 days post-insult. The number of activated, phagocytic macrophages also increased significantly in CC and ECL. HI also increased pro-inflammatory cytokine expression, including TNFα at P12 and IL-1β at P17 and P30. Interestingly, the anti-inflammatory cytokine IL-1ra was transiently upregulated at P12. Early microglial activation, often associated with an M2 immunomodulatory phenotype, is considered neuroprotective during early brain repair^41–46^. However, persistent microgliosis and sustained pro-inflammatory cytokine expression are linked to oligodendrocyte and neuronal injury^47^. In line with human and animal data, glial cells and neurons in our model appeared to have released inflammatory cytokines that contributed to ongoing neuroinflammation^48–53^. Reactive astrocytes, infiltrating the injured brain after HI, further amplified the inflammatory response^54–56^. This chronic inflammatory environment characterizes the tertiary phase of HIE injury, driven by both resident glia and infiltrating peripheral immune cells. Together, they create a toxic niche that hinders white matter maturation, neurogenesis, and synaptogenesis^57–59^. Sildenafil treatment significantly decreased the number of reactive astrocytes and activated microglia in the cortex and white matter. It normalized cytokine expression levels, including TNFα, IL-1β, and IL-1ra. These effects were most prominent with medium and high doses, consistent with previous findings showing a dose-dependent reduction in brain injury and improved neurological outcomes^19,20^. These data suggest that sildenafil may target the tertiary phase of injury, promoting neurorestoration. Similar anti-inflammatory effects of sildenafil have been observed in adult models of multiple sclerosis, stroke, and hepatic encephalopathy, where it reduced microglial/macrophage activation and pro-inflammatory cytokine release^23,25,29,60^.

Chronic neuroinflammation contributes to white matter injury by promoting axonal and myelin degeneration^61–64^. Reactive astrocytosis impairs oligodendrocyte maturation^65^, and elevated TNFα and IL-1β levels can directly induce oligodendrocyte apoptosis^59,66–69^. In our model, persistent neuroinflammation coincided with a reduction in thickness of the CC and ECL by P30. This finding is particularly relevant, since oligodendrocytes are highly susceptible to HI^70^ and are responsible for myelination during the tertiary phase. White matter injury occurs in nearly one-quarter of term neonates with HIE^71^ and is associated with adverse long-term neurodevelopmental outcomes^72,73^. Sildenafil treatment restored thickness of the CC and ECL and improved oligodendrocyte populations in a dose-dependent manner. Medium and high doses increased both total (Olig2 +) and mature (CC1 +) oligodendrocyte counts in the CC and ECL, but not immature ones (Olig2 + /CC1-). These findings indicate that sildenafil may promote oligodendrogenesis and support remyelination. Previous studies in adult models of stroke, multiple sclerosis, and diabetes support this hypothesis, demonstrating that sildenafil enhances oligodendrocyte regeneration, increases myelin thickness, and protects myelinated axons by modulating immune responses^24,28,29,74^. Sildenafil’s effects appear context-dependent, promoting oligodendrocyte maturation primarily under hypoxic-ischemic conditions. Its anti-inflammatory and pro-survival actions may create a permissive environment for oligodendrocyte maturation and myelin repair during injury, whereas in the absence of HI, these pathways are likely insufficiently activated to elicit further enhancement.

In addition to its effects on glial cells, HI reduced the expression of key neuronal and synaptic proteins, including NeuN and synaptophysin. Sildenafil restored their levels to those comparable to sham controls, which suggests neuronal preservation and synaptic repair. These results align with our prior data^19^ and with studies in adult disease models^75–77^ that report enhanced neuronal survival and increased synaptic protein expression following sildenafil treatment. Furthermore, sildenafil has exhibited notable anti-apoptotic effects. While HI induced a marked increase in apoptosis at P12, sildenafil treatment attenuated this response. Together, these results support both a neuroprotective and neurorestorative role for sildenafil, in agreement with prior studies demonstrating its ability to reduce apoptosis in neurons and oligodendrocytes^22,78–83^. Additional immunohistochemistry is required in our model to pinpoint the cell types affected by apoptosis and to determine whether sildenafil selectively mitigated apoptotic injury in oligodendrocytes or neurons.

Mechanistically, sildenafil may exert these effects via modulation of the PI3K/AKT/mTOR signaling pathway^84^. We observed that p-AKT levels were reduced following HI but were restored by sildenafil treatment. This restoration likely contributed to the anti-inflammatory and reparative effects of sildenafil, supporting white matter recovery^85^. This restoration is particularly relevant, since constitutive AKT activation has been shown to enhance myelin production^86^.

Finally, while neuroinflammation is necessary for repair, its persistence can be harmful. Modulating rather than eliminating this response is key. Previous studies using inhaled nitric oxide or sildenafil demonstrated reduced white matter inflammation and enhanced oligodendrocyte maturation in neonatal models^87,88^. Our findings support the therapeutic potential of sildenafil during the tertiary phase, provided that improvements in neurodevelopmental outcomes are confirmed^89,90^.

While our findings provide strong evidence for the neuroprotective and neurorestorative effects of sildenafil in a rat model of term neonatal HIE, several limitations should be considered. First, the present study focused on short- and intermediate-term histological and molecular outcomes up to P30; long-term neurodevelopmental and behavioral assessments are needed to determine whether these structural improvements translate into functional recovery. Second, we assessed the thickness of the corpus callosum (CC) and the left external capsule (ECL) as a morphometric proxy for white matter integrity and myelination. We acknowledge that myelin-specific staining (e.g. myelin basic protein, MBP, or Luxol Fast Blue) would provide a more direct assessment of myelin content; however, these stains were not available for the present dataset. The thickness measurements were therefore used as an established histological indicator of white matter atrophy or preservation in this model. Future work should include myelin-specific staining to further confirm these findings. Third, our interpretation of the PI3K/AKT/mTOR pathway modulation as a potential mechanism was based solely on pAKT (Ser473) expression normalized to β-actin, as total AKT data were not available. Ser473 phosphorylation is a well-established marker of AKT activation in neuroprotection studies; however, assessing additional sites, such as Thr308, would provide complementary insights into upstream signaling mechanisms. Establishing causality will require studies using selective inhibitors of mTORC1 and mTORC2. Further work should further characterize AKT activation following HI and sildenafil treatment and examine downstream mTOR targets and their crosstalk with inflammatory signaling to clarify the therapeutic actions of sildenafil. Fourth, our study was limited to male rats; given known sex-related differences in brain injury and repair, future research should evaluate sex-specific responses to HI and sildenafil treatment. Last, validation in large animal models is essential, along with investigation into optimal dosing, timing, and duration of treatment for potential clinical translation.

In conclusion, sildenafil treatment after HI significantly attenuated neuroinflammation, reduced glial activation and pro-inflammatory cytokine expression, and improved white matter integrity and markers of neurogenesis. These reparative effects were associated with restoration p-AKT levels and thus involvement of mTOR signaling. By modulating the inflammatory environment, sildenafil appears to promote a more favorable niche for the restoration of neurodevelopmental processes such as myelination, neurogenesis, and synaptogenesis. Further exploration of sildenafil’s impact on long-term myelination, neurogenesis, and functional outcomes will be critical to advancing its potential clinical translation for neonatal HIE.

## Data Availability

All data generated during this study are included in this published article.

## Abbreviations

HI: Hypoxia–ischemia
HIE: Hypoxic-ischemic encephalopathy
cGMP: Cyclic guanosine monophosphate
P: Postnatal
PDE5: Phosphodiesterase type 5 inhibitor
mTOR: Mechanistic target of rapamycin
Olig2: Oligodendrocyte transcription factor 2
TH: Therapeutic hypothermia

## References

[1] Kurinczuk, J. J., White-Koning, M. & Badawi, N. Epidemiology of neonatal encephalopathy and hypoxic-ischaemic encephalopathy. *Early Hum. Dev.* **86**, 329–338 (2010). 20554402 10.1016/j.earlhumdev.2010.05.010

[2] Tagin, M. A., Woolcott, C. G., Vincer, M. J., Whyte, R. K. & Stinson, D. A. Hypothermia for neonatal hypoxic ischemic encephalopathy: An updated systematic review and meta-analysis. *Arch. Pediatr. Adolesc. Med.* **166**, 558–566 (2012). 22312166 10.1001/archpediatrics.2011.1772

[3] Edwards, A. D. et al. Neurological outcomes at 18 months of age after moderate hypothermia for perinatal hypoxic ischaemic encephalopathy: Synthesis and meta-analysis of trial data. *BMJ* **340**, c363 (2010). 20144981 10.1136/bmj.c363 PMC2819259

[4] Azzopardi, D. V. et al. Moderate hypothermia to treat perinatal asphyxial encephalopathy. *N. Engl. J. Med.* **361**, 1349–1358 (2009). 19797281 10.1056/NEJMoa0900854

[5] Johnston, M. V., Trescher, W. H., Ishida, A. & Nakajima, W. Neurobiology of hypoxic-ischemic injury in the developing brain. *Pediatr. Res.* **49**, 735–741 (2001). 11385130 10.1203/00006450-200106000-00003

[6] Bennet, L., Roelfsema, V., Pathipati, P., Quaedackers, J. S. & Gunn, A. J. Relationship between evolving epileptiform activity and delayed loss of mitochondrial activity after asphyxia measured by near-infrared spectroscopy in preterm fetal sheep. *J. Physiol.* **572**, 141–154 (2006). 16484298 10.1113/jphysiol.2006.105197 PMC1779651

[7] Gunn, A. J., Gunn, T. R., de Haan, H. H., Williams, C. E. & Gluckman, P. D. Dramatic neuronal rescue with prolonged selective head cooling after ischemia in fetal lambs. *J. Clin. Investig.* **99**, 248–256 (1997). 9005993 10.1172/JCI119153 PMC507792

[8] Fleiss, B. & Gressens, P. Tertiary mechanisms of brain damage: A new hope for treatment of cerebral palsy?. *Lancet Neurol.* **11**, 556–566 (2012). 22608669 10.1016/S1474-4422(12)70058-3

[9] Benjelloun, N., Renolleau, S., Represa, A., Ben-Ari, Y. & Charriaut-Marlangue, C. Inflammatory responses in the cerebral cortex after ischemia in the P7 neonatal Rat. *Stroke* **30**, 1916–1923 (1999). 10471445 10.1161/01.str.30.9.1916

[10] Villapol, S., Gelot, A., Renolleau, S. & Charriaut-Marlangue, C. Astrocyte responses after neonatal ischemia: The yin and the yang. *Neuroscientist* **14**, 339–344 (2008). 18612085 10.1177/1073858408316003

[11] Pimentel, V. C. et al. Adenosine deaminase activity, lipid peroxidation and astrocyte responses in the cerebral cortex of rats after neonatal hypoxia ischemia. *Int. J. Dev. Neurosci.* **27**, 857–862 (2009). 19559780 10.1016/j.ijdevneu.2009.06.003

[12] Mallard, C., Tremblay, M. E. & Vexler, Z. S. Microglia and neonatal brain injury. *Neuroscience* **405**, 68–76 (2019). 29352997 10.1016/j.neuroscience.2018.01.023 PMC6790108

[13] Bennet, L. et al. Cell therapy for neonatal hypoxia-ischemia and cerebral palsy. *Ann. Neurol.* **71**, 589–600 (2012). 22522476 10.1002/ana.22670

[14] Douglas-Escobar, M. & Weiss, M. D. Hypoxic-ischemic encephalopathy: A review for the clinician. *JAMA Pediatr.* **169**, 397–403 (2015). 25685948 10.1001/jamapediatrics.2014.3269

[15] Bednar, M. M. The role of sildenafil in the treatment of stroke. *Curr. Opin. Investig. Drugs* **9**, 754–759 (2008). 18600581

[16] Zhang, L. et al. Functional recovery in aged and young rats after embolic stroke: Treatment with a phosphodiesterase type 5 inhibitor. *Stroke* **36**, 847–852 (2005). 15746452 10.1161/01.STR.0000158923.19956.73

[17] Steinhorn, R. H. et al. Intravenous sildenafil in the treatment of neonates with persistent pulmonary hypertension. *J. Pediatr.* **155**, 841-847.e841 (2009). 19836028 10.1016/j.jpeds.2009.06.012

[18] Shah, P. S. & Ohlsson, A. Sildenafil for pulmonary hypertension in neonates. *Cochrane Database Syst. Rev.*10.1002/14651858.CD005494.pub4 (2011). 21833954 10.1002/14651858.CD005494.pub3

[19] Yazdani, A. et al. Sildenafil improves brain injury recovery following term neonatal hypoxia-ischemia in male rat pups. *Dev. Neurosci.* **38**, 251–263 (2016). 27614933 10.1159/000448327

[20] Jung, S. et al. Sildenafil improves functional and structural outcome of retinal injury following term neonatal hypoxia-ischemia. *Investig. Ophthalmol. Vis. Sci.* **57**, 4306–4314 (2016). 27552408 10.1167/iovs.16-19385

[21] Charriaut-Marlangue, C. et al. Sildenafil mediates blood-flow redistribution and neuroprotection after neonatal hypoxia-ischemia. *Stroke* **45**, 850–856 (2014). 24473179 10.1161/STROKEAHA.113.003606

[22] Duarte-Silva, E. et al. Sildenafil ameliorates EAE by decreasing apoptosis in the spinal cord of C57BL/6 mice. *J. Neuroimmunol.* **321**, 125–137 (2018). 29957383 10.1016/j.jneuroim.2018.06.002

[23] Pifarre, P. et al. Sildenafil (Viagra) ameliorates clinical symptoms and neuropathology in a mouse model of multiple sclerosis. *Acta Neuropathol.* **121**, 499–508 (2011). 21234581 10.1007/s00401-010-0795-6

[24] Pifarré, P. et al. Phosphodiesterase 5 inhibition at disease onset prevents experimental autoimmune encephalomyelitis progression through immunoregulatory and neuroprotective actions. *Exp. Neurol.* **251**, 58–71 (2014). 24211383 10.1016/j.expneurol.2013.10.021

[25] Agusti, A. et al. Sildenafil reduces neuroinflammation in cerebellum, restores GABAergic tone, and improves motor in-coordination in rats with hepatic encephalopathy. *CNS Neurosci. Ther.* **23**, 386–394 (2017). 28296282 10.1111/cns.12688 PMC6492705

[26] Raposo, C. et al. Sildenafil (Viagra) protective effects on neuroinflammation: The role of iNOS/NO system in an inflammatory demyelination model. *Mediators Inflamm.* **2013**, 321460 (2013). 23970812 10.1155/2013/321460 PMC3736464

[27] Zhang, J. et al. Phosphodiesterase-5 inhibitor sildenafil prevents neuroinflammation, lowers beta-amyloid levels and improves cognitive performance in APP/PS1 transgenic mice. *Behav. Brain Res.* **250**, 230–237 (2013). 23685322 10.1016/j.bbr.2013.05.017

[28] Zhang, R. L. et al. Sildenafil enhances neurogenesis and oligodendrogenesis in ischemic brain of middle-aged mouse. *PLoS ONE* **7**, e48141 (2012). 23118941 10.1371/journal.pone.0048141 PMC3485244

[29] Nunes, A. K., Rapôso, C., Luna, R. L., Cruz-Höfling, M. A. & Peixoto, C. A. Sildenafil (Viagra®) down regulates cytokines and prevents demyelination in a cuprizone-induced MS mouse model. *Cytokine* **60**, 540–551 (2012). 22749439 10.1016/j.cyto.2012.06.011

[30] Percie du Sert, N. et al. The ARRIVE guidelines 2.0: Updated guidelines for reporting animal research. *PLoS Biol.* **18**, e3000410 (2020). 32663219 10.1371/journal.pbio.3000410 PMC7360023

[31] Northington, F. J. Brief update on animal models of hypoxic-ischemic encephalopathy and neonatal stroke. *Ilar J.* **47**, 32–38 (2006). 16391429 10.1093/ilar.47.1.32

[32] Rice, J. E. 3rd., Vannucci, R. C. & Brierley, J. B. The influence of immaturity on hypoxic-ischemic brain damage in the rat. *Ann. Neurol.* **9**, 131–141 (1981). 7235629 10.1002/ana.410090206

[33] Patel, S. D., Pierce, L., Ciardiello, A. J. & Vannucci, S. J. Neonatal encephalopathy: Pre-clinical studies in neuroprotection. *Biochem. Soc. Trans.* **42**, 564–568 (2014). 24646279 10.1042/BST20130247

[34] Vannucci, R. C. & Vannucci, S. J. Perinatal hypoxic-ischemic brain damage: Evolution of an animal model. *Dev. Neurosci.* **27**, 81–86 (2005). 16046840 10.1159/000085978

[35] Recker, R. et al. Rodent neonatal bilateral carotid artery occlusion with hypoxia mimics human hypoxic-ischemic injury. *J. Cereb. Blood Flow Metab.* **29**, 1305–1316 (2009). 19436315 10.1038/jcbfm.2009.56

[36] Quinn, R. Comparing rat’s to human’s age: How old is my rat in people years?. *Nutrition* **21**, 775–777 (2005). 15925305 10.1016/j.nut.2005.04.002

[37] Vannucci, R. C. Experimental models of perinatal hypoxic-ischemic brain damage. *APMIS Suppl.* **40**, 89–95 (1993). 8311995

[38] Vannucci, R. C. et al. Rat model of perinatal hypoxic-ischemic brain damage. *J. Neurosci. Res.* **55**, 158–163 (1999). 9972818 10.1002/(SICI)1097-4547(19990115)55:2<158::AID-JNR3>3.0.CO;2-1

[39] Walker, D. K. et al. Pharmacokinetics and metabolism of sildenafil in mouse, rat, rabbit, dog and man. *Xenobiotica* **29**, 297–310 (1999). 10219969 10.1080/004982599238687

[40] Schneider, C. A., Rasband, W. S. & Eliceiri, K. W. NIH Image to ImageJ: 25 years of image analysis. *Nat. Methods* **9**, 671–675 (2012). 22930834 10.1038/nmeth.2089 PMC5554542

[41] Chen, C. Y. et al. Hypoxic preconditioning suppresses glial activation and neuroinflammation in neonatal brain insults. *Mediators Inflamm.* **2015**, 632592 (2015). 26273140 10.1155/2015/632592 PMC4530271

[42] McRae, A., Gilland, E., Bona, E. & Hagberg, H. Microglia activation after neonatal hypoxic-ischemia. *Brain Res. Dev. Brain Res.* **84**, 245–252 (1995). 7743644 10.1016/0165-3806(94)00177-2

[43] Kreutzberg, G. W. Microglia: A sensor for pathological events in the CNS. *Trends Neurosci.* **19**, 312–318 (1996). 8843599 10.1016/0166-2236(96)10049-7

[44] Ekdahl, C. T., Kokaia, Z. & Lindvall, O. Brain inflammation and adult neurogenesis: The dual role of microglia. *Neuroscience* **158**, 1021–1029 (2009). 18662748 10.1016/j.neuroscience.2008.06.052

[45] Butovsky, O. et al. Microglia activated by IL-4 or IFN-gamma differentially induce neurogenesis and oligodendrogenesis from adult stem/progenitor cells. *Mol. Cell Neurosci.* **31**, 149–160 (2006). 16297637 10.1016/j.mcn.2005.10.006

[46] Krady, J. K. et al. Ciliary neurotrophic factor and interleukin-6 differentially activate microglia. *J. Neurosci. Res.* **86**, 1538–1547 (2008). 18214991 10.1002/jnr.21620

[47] Kaur, C., Rathnasamy, G. & Ling, E. A. Roles of activated microglia in hypoxia induced neuroinflammation in the developing brain and the retina. *J. Neuroimmune Pharmacol.* **8**, 66–78 (2013). 22367679 10.1007/s11481-012-9347-2

[48] Aly, H., Khashaba, M. T., El-Ayouty, M., El-Sayed, O. & Hasanein, B. M. IL-1beta, IL-6 and TNF-alpha and outcomes of neonatal hypoxic ischemic encephalopathy. *Brain Dev.* **28**, 178–182 (2006). 16181755 10.1016/j.braindev.2005.06.006

[49] Liu, J. & Feng, Z. C. Increased umbilical cord plasma interleukin-1 beta levels was correlated with adverse outcomes of neonatal hypoxic-ischemic encephalopathy. *J. Trop. Pediatr.* **56**, 178–182 (2010). 19822562 10.1093/tropej/fmp098

[50] Silveira, R. C. & Procianoy, R. S. Interleukin-6 and tumor necrosis factor-alpha levels in plasma and cerebrospinal fluid of term newborn infants with hypoxic-ischemic encephalopathy. *J. Pediatr.* **143**, 625–629 (2003). 14615734 10.1067/S0022-3476(03)00531-6

[51] Li, S. J. et al. The role of TNF-α, IL-6, IL-10, and GDNF in neuronal apoptosis in neonatal rat with hypoxic-ischemic encephalopathy. *Eur. Rev. Med. Pharmacol. Sci.* **18**, 905–909 (2014). 24706318

[52] Szaflarski, J., Burtrum, D. & Silverstein, F. S. Cerebral hypoxia-ischemia stimulates cytokine gene expression in perinatal rats. *Stroke* **26**, 1093–1100 (1995). 7762028 10.1161/01.str.26.6.1093

[53] Hagberg, H. et al. Enhanced expression of interleukin (IL)-1 and IL-6 messenger RNA and bioactive protein after hypoxia-ischemia in neonatal rats. *Pediatr. Res.* **40**, 603–609 (1996). 8888290 10.1203/00006450-199610000-00015

[54] Ohno, M., Aotani, H. & Shimada, M. Glial responses to hypoxic/ischemic encephalopathy in neonatal rat cerebrum. *Brain Res. Dev. Brain Res.* **84**, 294–298 (1995). 7743650 10.1016/0165-3806(94)00194-5

[55] Rossi, D. J., Brady, J. D. & Mohr, C. Astrocyte metabolism and signaling during brain ischemia. *Nat. Neurosci.* **10**, 1377–1386 (2007). 17965658 10.1038/nn2004 PMC8906499

[56] Takano, T., Oberheim, N., Cotrina, M. L. & Nedergaard, M. Astrocytes and ischemic injury. *Stroke* **40**, S8-12 (2009). 19064795 10.1161/STROKEAHA.108.533166 PMC2653262

[57] Juul, S. E. & Ferriero, D. M. Pharmacologic neuroprotective strategies in neonatal brain injury. *Clin. Perinatol.* **41**, 119–131 (2014). 24524450 10.1016/j.clp.2013.09.004 PMC3929237

[58] Dixon, B. J., Reis, C., Ho, W. M., Tang, J. & Zhang, J. H. Neuroprotective strategies after neonatal hypoxic ischemic encephalopathy. *Int. J. Mol. Sci.* **16**, 22368–22401 (2015). 26389893 10.3390/ijms160922368 PMC4613313

[59] Li, B., Concepcion, K., Meng, X. & Zhang, L. Brain-immune interactions in perinatal hypoxic-ischemic brain injury. *Prog. Neurobiol.* **159**, 50–68 (2017). 29111451 10.1016/j.pneurobio.2017.10.006 PMC5831511

[60] Moretti, R. et al. Sildenafil, a cyclic GMP phosphodiesterase inhibitor, induces microglial modulation after focal ischemia in the neonatal mouse brain. *J. Neuroinflamm.* **13**, 95 (2016). 10.1186/s12974-016-0560-4 PMC4850658 27126393

[61] Peixoto, C. A., Nunes, A. K. & Garcia-Osta, A. Phosphodiesterase-5 inhibitors: Action on the signaling pathways of neuroinflammation, neurodegeneration, and cognition. *Mediat. Inflamm.* **2015**, 940207 (2015). 10.1155/2015/940207 PMC4681825 26770022

[62] Johnson, V. E. et al. Inflammation and white matter degeneration persist for years after a single traumatic brain injury. *Brain* **136**, 28–42 (2013). 23365092 10.1093/brain/aws322 PMC3562078

[63] Kempuraj, D. et al. Neuroinflammation induces neurodegeneration. *J. Neurol. Neurosurg. Spine* **1**(1), 1003 (2016). 28127589 PMC5260818

[64] Fuster-Matanzo, A., Llorens-Martín, M., Hernández, F. & Avila, J. Role of neuroinflammation in adult neurogenesis and Alzheimer disease: Therapeutic approaches. *Mediators Inflamm.* **2013**, 260925 (2013). 23690659 10.1155/2013/260925 PMC3649701

[65] Shiow, L. R. et al. Reactive astrocyte COX2-PGE2 production inhibits oligodendrocyte maturation in neonatal white matter injury. *Glia* **65**, 2024–2037 (2017). 28856805 10.1002/glia.23212 PMC5753598

[66] Li, J. et al. Tumor necrosis factor alpha mediates lipopolysaccharide-induced microglial toxicity to developing oligodendrocytes when astrocytes are present. *J. Neurosci.* **28**, 5321–5330 (2008). 18480288 10.1523/JNEUROSCI.3995-07.2008 PMC2677805

[67] Deng, Y. et al. Astrocyte-derived proinflammatory cytokines induce hypomyelination in the periventricular white matter in the hypoxic neonatal brain. *PLoS ONE* **9**, e87420 (2014). 24498101 10.1371/journal.pone.0087420 PMC3909103

[68] Guadagno, J., Swan, P., Shaikh, R. & Cregan, S. P. Microglia-derived IL-1β triggers p53-mediated cell cycle arrest and apoptosis in neural precursor cells. *Cell Death Dis* **6**, e1779 (2015). 26043079 10.1038/cddis.2015.151 PMC4669832

[69] Xie, D. et al. IL-1β induces hypomyelination in the periventricular white matter through inhibition of oligodendrocyte progenitor cell maturation via FYN/MEK/ERK signaling pathway in septic neonatal rats. *Glia* **64**, 583–602 (2016). 26678483 10.1002/glia.22950

[70] Wyatt, J. S. Mechanisms of brain injury in the newborn. *Eye (Lond)* **21**, 1261–1263 (2007). 17914428 10.1038/sj.eye.6702848

[71] Li, A. M. et al. White matter injury in term newborns with neonatal encephalopathy. *Pediatr. Res.* **65**, 85–89 (2009). 18787422 10.1203/PDR.0b013e31818912d2

[72] Miller, S. P. et al. Early brain injury in premature newborns detected with magnetic resonance imaging is associated with adverse early neurodevelopmental outcome. *J. Pediatr.* **147**, 609–616 (2005). 16291350 10.1016/j.jpeds.2005.06.033

[73] Woodward, L. J., Anderson, P. J., Austin, N. C., Howard, K. & Inder, T. E. Neonatal MRI to predict neurodevelopmental outcomes in preterm infants. *N. Engl. J. Med.* **355**, 685–694 (2006). 16914704 10.1056/NEJMoa053792

[74] Wang, L. et al. Phosphodiesterase-5 is a therapeutic target for peripheral neuropathy in diabetic mice. *Neuroscience* **193**, 399–410 (2011). 21820491 10.1016/j.neuroscience.2011.07.039 PMC3391742

[75] Agostino, P. V., Plano, S. A. & Golombek, D. A. Sildenafil accelerates reentrainment of circadian rhythms after advancing light schedules. *Proc. Natl. Acad. Sci. U. S. A.* **104**, 9834–9839 (2007). 17519328 10.1073/pnas.0703388104 PMC1887561

[76] Venkat, P. et al. Sildenafil treatment of vascular dementia in aged rats. *Neurochem. Int.* **127**, 103–112 (2019). 30592970 10.1016/j.neuint.2018.12.015

[77] Mulhall, J. P. et al. The functional and structural consequences of cavernous nerve injury are ameliorated by sildenafil citrate. *J. Sex Med.* **5**, 1126–1136 (2008). 18331274 10.1111/j.1743-6109.2008.00794.x

[78] Zhang, Q. et al. The effects of phosphodiesterase-5 inhibitor sildenafil against post-resuscitation myocardial and intestinal microcirculatory dysfunction by attenuating apoptosis and regulating microRNAs expression: Essential role of nitric oxide syntheses signaling. *J. Transl. Med.* **13**, 177 (2015). 26040988 10.1186/s12967-015-0550-9 PMC4467614

[79] Das, A. et al. Sildenafil (Viagra) sensitizes prostate cancer cells to doxorubicin-mediated apoptosis through CD95. *Oncotarget* **7**, 4399–4413 (2016). 26716643 10.18632/oncotarget.6749 PMC4826214

[80] Das, A., Xi, L. & Kukreja, R. C. Phosphodiesterase-5 inhibitor sildenafil preconditions adult cardiac myocytes against necrosis and apoptosis. Essential role of nitric oxide signaling. *J. Biol. Chem.* **280**, 12944–12955 (2005). 15668244 10.1074/jbc.M404706200

[81] Mulhall, J. P. et al. Sildenafil citrate improves erectile function after castration in a rat model. *BJU Int.* **113**, 656–661 (2014). 23773301 10.1111/bju.12175

[82] Duarte-Silva, E. & Peixoto, C. A. Molecular mechanisms of phosphodiesterase-5 inhibitors on neuronal apoptosis. *DNA Cell Biol.* **37**, 861–865 (2018). 30234372 10.1089/dna.2018.4410

[83] Hutchings, D. C., Anderson, S. G., Caldwell, J. L. & Trafford, A. W. Phosphodiesterase-5 inhibitors and the heart: Compound cardioprotection?. *Heart* **104**, 1244–1250 (2018). 29519873 10.1136/heartjnl-2017-312865 PMC6204975

[84] Narayanan, S. P., Flores, A. I., Wang, F. & Macklin, W. B. Akt signals through the mammalian target of rapamycin pathway to regulate CNS myelination. *J. Neurosci.* **29**, 6860–6870 (2009). 19474313 10.1523/JNEUROSCI.0232-09.2009 PMC2757755

[85] Srivastava, I. N., Shperdheja, J., Baybis, M., Ferguson, T. & Crino, P. B. mTOR pathway inhibition prevents neuroinflammation and neuronal death in a mouse model of cerebral palsy. *Neurobiol. Dis.* **85**, 144–154 (2016). 26459113 10.1016/j.nbd.2015.10.001

[86] Flores, A. I. et al. Constitutively active Akt induces enhanced myelination in the CNS. *J. Neurosci.* **28**, 7174–7183 (2008). 18614687 10.1523/JNEUROSCI.0150-08.2008 PMC4395496

[87] Pham, H. et al. Impact of inhaled nitric oxide on white matter damage in growth-restricted neonatal rats. *Pediatr. Res.* **77**, 563–569 (2015). 25580736 10.1038/pr.2015.4

[88] Pham, H. et al. Inhaled NO prevents hyperoxia-induced white matter damage in neonatal rats. *Exp. Neurol.* **252**, 114–123 (2014). 24322053 10.1016/j.expneurol.2013.11.025

[89] Wintermark, P. et al. Feasibility and safety of sildenafil to repair brain injury secondary to birth asphyxia (SANE-01): A randomized, double-blind, placebo-controlled phase Ib clinical trial. *J. Pediatr.* **266**, 113879 (2024). 38142044 10.1016/j.jpeds.2023.113879

[90] Wintermark, P. et al. Testing higher doses of sildenafil to repair brain injury secondary to birth asphyxia: An open-label dose-finding phase 1b clinical trial (SANE-02). *J. Pediatr.* **285**, 114701 (2025). 40562301 10.1016/j.jpeds.2025.114701

